# Porcine reproductive and respiratory syndrome virus nonstructural protein 2 contributes to NF-κB activation

**DOI:** 10.1186/1743-422X-9-83

**Published:** 2012-04-30

**Authors:** Ying Fang, Liurong Fang, Yang Wang, Yingying Lei, Rui Luo, Dang Wang, Huanchun Chen, Shaobo Xiao

**Affiliations:** 1 Division of Animal Infectious Diseases, State Key Laboratory of Agricultural Microbiology, College of Veterinary Medicine, Huazhong Agricultural University, 1 Shizishan street, Wuhan, 430070, People’s Republic of China

**Keywords:** Porcine reproductive and respiratory syndrome virus, Nonstructural protein 2, NF-κB

## Abstract

**Background:**

Nuclear factor-kappaB (NF-κB) is an inducible transcription factor that plays a key role in inflammation and immune responses, as well as in the regulation of cell proliferation and survival. Previous studies by our group and others have demonstrated that porcine reproductive and respiratory syndrome virus (PRRSV) infection could activate NF-κB in MARC-145 cells and alveolar macrophages. The nucleocapsid (N) protein was identified as an NF-κB activator among the structural proteins encoded by PRRSV; however, it remains unclear whether the nonstructural proteins (Nsps) contribute to NF-κB activation. In this study, we identified which Nsps can activate NF-κB and investigated the potential mechanism(s) by which they act.

**Results:**

By screening the individual Nsps of PRRSV strain WUH3, Nsp2 exhibited great potential to activate NF-κB in MARC-145 and HeLa cells. Overexpression of Nsp2 induced IκBα degradation and nuclear translocation of NF-κB. Furthermore, Nsp2 also induced NF-κB-dependent inflammatory factors, including interleukin (IL)-6, IL-8, COX-2, and RANTES. Compared with the Nsp2 of the classical PRRSV strain, the Nsp2 of highly pathogenic PRRSV (HP-PRRSV) strains that possess a 30 amino acid (aa) deletion in Nsp2 displayed greater NF-κB activation. However, the 30-aa deletion was demonstrated to not be associated with NF-κB activation. Further functional domain analyses revealed that the hypervariable region (HV) of Nsp2 was essential for NF-κB activation.

**Conclusions:**

Taken together, these data indicate that PRRSV Nsp2 is a multifunctional protein participating in the modulation of host inflammatory response, which suggests an important role of Nsp2 in pathogenesis and disease outcomes.

## Background

Porcine reproductive and respiratory syndrome (PRRS) is characterized by severe reproductive failure in sows, and respiratory distress in piglets and growing pigs, and continues to be the most economically significant disease in the swine industry [1,2]. The causative agent, PRRS virus (PRRSV), is an enveloped, single-stranded positive sense RNA virus containing a genome of approximately 15 kb [3]. The 5’ two-thirds of the viral genome is occupied by overlapping open reading frames 1a (ORF1a) and 1b, which encode nonstructural polyproteins (Nsp), pp1a and pp1ab, respectively [4,5]. These polyproteins are proteolytically processed into 14 nonstructural proteins (Nsp1α, Nsp1β, and Nsp2 to 12) [6-8]. In 2006, an atypical PRRS outbreak in China was caused by an emerging highly-pathogenic PRRSV (HP-PRRSV) strain with a discontinuous deletion of 30-amino acid in the Nsp2 coding region in comparison with classical PRRSV strains [9,10]. Since then, the HP-PRRSV strain, which caused a more severe pneumonia and higher mortality than the classical PRRSV, has become the dominant strain found in China [11,12]. The immense genetic variability in the coding region of Nsp2 suggests that Nsp2 may play a limited role in viral replication but likely to play a very important part in modulating virus pathogenesis and the inflammation response in pigs during infection [13-16].

NF-κB is a family of inducible transcription factors involving pathogen- or cytokine-induced immune and inflammatory responses, as well as cell proliferation and survival [17-20]. The members of the NF-κB family in mammalian cells include p50/p105 (NFκB1), p65 (RelA), p52/p100 (NFκB2), c-Rel, and RelB. All of these proteins share a conserved 300-amino acid region known as the Rel homology domain that is responsible for DNA binding, dimerization, and nuclear translocation of NF-κB. In most cells, Rel family members form hetero- and homodimers with distinct specificities in various combinations [21,22]. Classical NF-κB exists as heterodimers consisting of a 50-kDa subunit (p50) and a 65-kDa subunit (p65) [23]. Under normal physiological conditions, NF-κB is sequestered in the cytoplasm as inactive complexes by its interaction with a member of the inhibitory kappa B (IκB) family. When stimulated with a wide range of proinflammatory stimuli, the IκB proteins are phosphorylated by IκB kinase (IKK) and degraded in proteasomes [24]. The subunit of NF-κB p65 is then phosphorylated while exposing its nuclear localization signal sequence (NLS), leading to nuclear translocation and subsequent binding of NF-κB to DNA regulatory elements of the target genes involved in various biological functions [6,21-23,25].

Many viruses encode proteins that activate or modulate NF-κB signalling pathways for their own advantage [26]. For example, UL37 tegument protein, encoded by herpes simplex virus 1 (HSV-1) interacts with tumour necrosis factor (TNF) receptor-associated factor 6 (TRAF6), resulting in the NF-κB activation [27]. Similarly, NF-κB activation induced by Hepatitis C virus (HCV) core protein and Epstein–Barr virus latent membrane protein 1 (LMP1) is important for infected cell survival and persistent viral infection [28,29]. Previous studies of PRRSV have demonstrated that the nucleocapsid (N) protein was a NF-κB activator [30-32]; however, it remains unclear whether the PRRSV Nsps contribute to NF-κB activation. The current study demonstrated that PRRSV Nsp2 alone could activate the NF-κB signalling pathway and the potential molecular mechanism(s) for NF-κB activation were also investigated.

## Results and discussion

### PRRSV Nsp2 activates NF-kB

**Figure 1:**
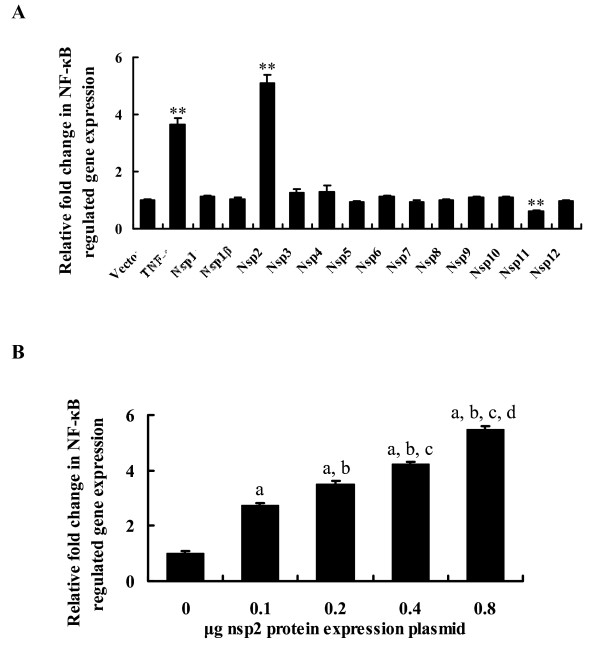
PRRSV Nsp2 activates NF-κB. (A) HeLa cells were co-transfected with the NF-κB-Luc reporter plasmid (0.2 μg), pRL-TK plasmid (0.05 μg), along with 0.6 μg of the indicated expression plasmid encoding PRRSV nonstructural protein. Selected cells were stimulated by 20 ng/ml TNF-α at 30 h post-transfection, and whole-cell extracts were prepared for the dual-luciferase activity at 6 h after this treatment. **P < 0.01 as compared with vector control. (B) An increasing quantities of indicated Nsp2 expression plasmid (0, 0.1, 0.2, 0.4, 0.8 μg) was co-transfected with pNF-κB-Luc and pRL-TK into HeLa cells. Cells were harvested at 36 h after transfection and analysed for luciferase activity. (a) *P <* 0.05 compared with the vector group, (b) *P <* 0.05 compared with 0.1 μg Nsp2 protein transfection group, (c) *P <* 0.05 compared with 0.2 μg Nsp2 protein transfection group, (d) *P <* 0.05 compared with 0.4 μg Nsp2 protein transfection group. Values for the samples were normalized using Renilla luciferase values and expressed as relative fold change in NF-κB-regulated gene expression compared with vector group, and each untreated empty vector control value was set as a basis level of 1. Data represent means of three replicates and results are representative of at least three independent experiments.

To identify the specific PRRSV Nsp(s) involved in NF-κB activation, all non-structural genes of PRRSV strain WUH3 [33] were screened for their capacities to activate NF-κB using a luciferase reporter assay. As shown in Figure 1A, overexpression of Nsp2 in HeLa cells potently induced NF-κB activation, while this activation was not observed in cells overexpressing other viral nonstructural proteins. To further confirm the effect of Nsp2 on NF-κB, HeLa cells were transfected with increasing amounts of Nsp2 expression plasmid and NF-κB luciferase activity was monitored at 36 h post-transfection. As shown in Figure 1B, a dose-dependent increase in luciferase reporter activity was observed. These data clearly indicated that Nsp2 was responsible for the induction of NF-κB activation among PRRSV nonstructural proteins. Similar results were obtained using MARC-145 cells overexpressing Nsp2 (data not shown).

### Nsp2 induced degradation of IκBα and nuclear translocation of NF-κB

**Figure 2:**
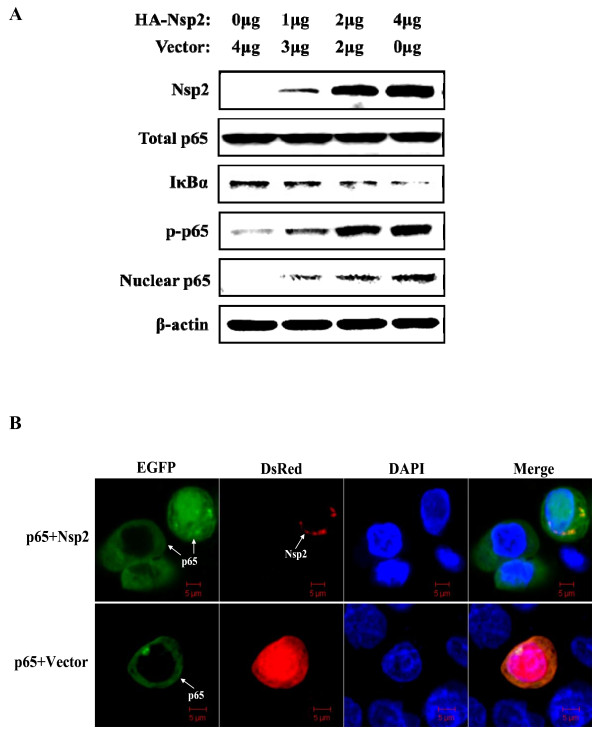
Degradation of IκBα and nuclear translocation of p65 are induced by PRRSV Nsp2. (A) HeLa cells were transfected with the indicated amount (0, 1, 2, 4 μg) of Nsp2 expression plasmid. Cytoplasmic and nuclear extracts were prepared at 48 h post-transfection and subjected to western blot analysis with antibodies specific for endogenous IκBα, p65 or p-p65. Mouse anti-HA was used to confirm the expression of HA-conjugated Nsp2 protein. Anti-β-actin was included as a control for sample loading. Western blot analyses were repeated in two independent experiments with similar results and a representative blot was shown. (B) HeLa cells were co-transfected with RFP-tagged Nsp2 expression plasmid (pDsRed-Nsp2) and the EGFP-tagged p65 expression plasmid (pEGFP-p65). Cells co-transfected with pDsRed-C1 and EGFP-tagged p65 served as a negative control. Cells were fixed and permeabilized at 36 h post-transfection. Cellular nuclei were counterstained with 1 μg/mL of DAPI (4',6-diamidino-2-phenylindole, dihydrochloride). Nuclear translocation of NF-κB was observed under an LSM-510 Meta confocal fluorescence microscope. Scale bar represents 5 μm.

Activation of NF-κB is usually characterized by degradation of IκBα after phosphorylation by IKK in response to many extracellular stimuli, which is followed by the phosphorylation of NF-κB subunit p65 (RelA) and nuclear translocation of NF-κB [19,22]. To investigate the potential mechanism(s) of NF-κB activation by Nsp2, we first examined the IκBα expression level in the cytoplasmic extracts using a western blot assay. As shown in Figure 2A, the IκBα protein degraded in a dose-dependent manner in Nsp2-transfected cells. To further characterize the mechanism, the phosphorylation and the nuclear translocation of the p65 subunit, as well as the total p65 in Nsp2 expression cells were determined. As shown in Figure 2A, the amount of phosphorylated p65 and nuclear p65 protein increased in a dose-dependent manner, while the amount of total p65 was unaltered. To further validate NF-κB nuclear translocation after Nsp2 overexpression, HeLa cells co-transfected with enhanced green fluorescent protein (EGFP)-p65 and red fluorescent protein (RFP)-Nsp2 fusion expression constructs were used to monitor the translocation of p65 by confocal fluorescence microscopy. As shown in Figure 2B, the p65 protein accumulated in the nucleus when co-expressed with Nsp2, while it was retained in the cytoplasm when co-expressed with the empty vector or expressed alone.

Overall, these results clearly demonstrated that Nsp2 induced NF-κB activation characterized by IκBα degradation and p65 nuclear translocation, which is consistent with a recent publication showing that PRRSV infection modulates NF-κB activation through IκBα degradation *in vitro*[31]. The observation that Nsp2 induced IκBα degradation raised the possibility that Nsp2 may have affected upstream processing for IκB phosphorylation from which the dissociation of the NF-κB**-**IκB complex and subsequent p65 nuclear translocation was dependent.

### PRRSV Nsp2 significantly enhanced NF-κB-regulated gene expression

**Figure 3:**
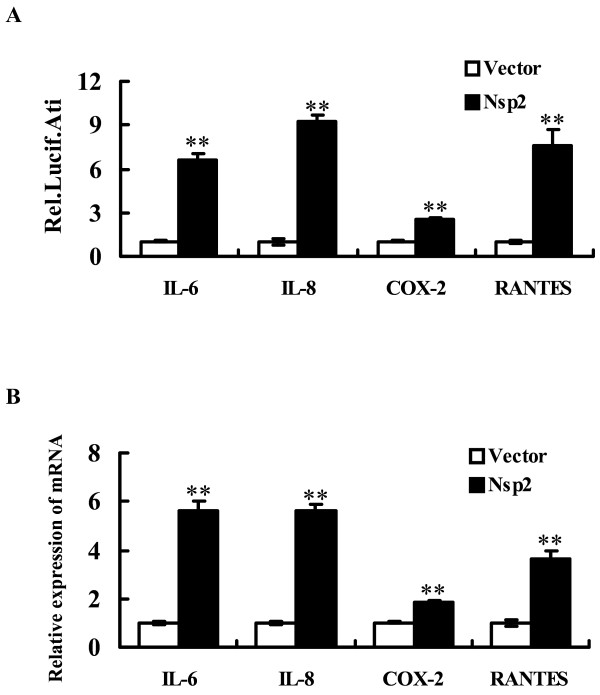
PRRSV Nsp2 significantly enhances NF-κB-regulated gene expression. (A) The wild-type Nsp2 (0.6 μg) and the empty vector were transfected into HeLa cells along with various reporter plasmids (0.2 μg) and pRL-TK (0.05 μg), respectively. Luciferase reporter assays were performed at 36 h post-transfection. The results are expressed as fold induction of IL-6, IL-8, COX-2, and RANTES promoter luciferase activity relative to the basal level. (B) HeLa cells were transfected with 1 μg of plasmid encoding Nsp2, or empty vector. After 36 h incubation, total RNA was extracted and the expression of IL-6, IL-8, COX-2, RANTES, and GAPDH genes were evaluated by real-time RT-PCR. Results are expressed as increasing mRNA levels relative to those in cells transfected with the empty vector and were normalized by using GAPDH housekeeping gene expression. All data represent the means and standard deviation of three independent experiments. **P < 0.01 as compared with vector control.

NF-κB is a critical transcription factor regulating the transcription and expression of many pro-inflammatory molecules, including certain adhesion molecules (ICAM-1), critical enzymes (for example, COX-2), most cytokines (for example, IL-1β, IL-6, and TNF-α), and chemokines (for example, IL-8 and RANTES) [19,34]. Previous studies have shown that PRRSV infection can induce several cytokines, such as IL-6, IL-8, IL-10, RANTES, and TNF-α, in porcine macrophages involved in the pulmonary inflammatory response [34,35]. To further investigate the possible biological impact of Nsp2 on pro-inflammatory molecules, a promoter luciferase reporter assay was used to determine if Nsp2 enhanced NF-κB-regulated target gene expression, including IL-6, IL-8, COX-2, and RANTES. As shown in Figure 3A, overexpression of Nsp2 enhanced the promoter activities of IL-6, IL-8, COX-2, and RANTES to varying degrees. To further confirm these results, the mRNA expression of these cytokines was detected by real-time reverse transcription-polymerase chain reaction (RT-PCR). As shown in Figure 3B, gene expression data were consistent with the promoter-driven luciferase reporter assay. Interestingly, as a result of treatment with the NF-κB inhibitor, BAY-117082, Nsp2 exhibited reduced ability to upregulate IL-6, IL-8, COX-2, and RANTES expression in a dose-dependent manner (data not shown). These observations suggest that Nsp2 stimulated cytokine expression via the NF-κB signalling pathway.

### The 30-amino acid deletion in the Nsp2 of HP-PRRSV has no effect on NF-κB activation

**Figure 4:**
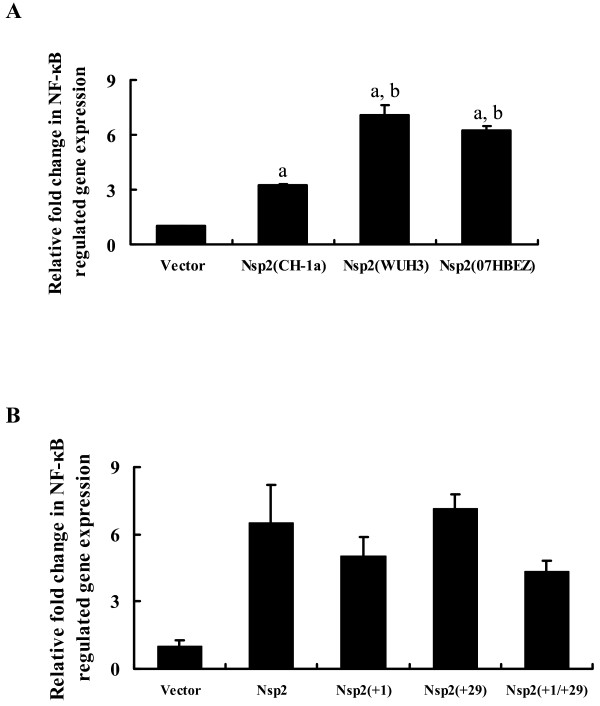
Amino acid deletion was not necessary for Nsp2 induced activation of NF-κB. (A) HeLa cells were co-transfected with the NF-κB-Luc reporter plasmid (0.2 μg), 0.05 μg of pRL-TK, and the designated Nsp2 expression plasmids (0.6 μg). An empty vector was used as a control. Cell extracts were collected at 36 h post-transfection and analysed for luciferase expression. (a) *P <* 0.05 compared with the vector group, (b) *P <* 0.05 compared with Nsp2 (CH-1a) protein transfection group. (B) Distinct insertion mutants or wild-type Nsp2 were transfected into HeLa cells together with NF-κB-Luc (0.2 μg) and pRL-TK (0.05 μg). Cell lysates were prepared at 36 h post-transfection and subjected to a dual-luciferase assay. There was no significant statistical difference between the wild-type Nsp2 and its mutants to activate NF-κB (*P* > 0.05).

Compared with the classical PRRSV strains, animals infected with HP-PRRSV typically show a more severe pneumonia. It is well known that NF-κB is an important transcription factor for mediating virus-induced inflammatory responses, such as severe lung inflammation and diseases [19,36]. Thus, we assessed the ability of Nsp2 from different PRRSV strains to activate NF-κB using a luciferase reporter assay. As shown in Figure 4A, Nsp2 of HP-PRRSV strains (WUH3 and 07HBEZ) could induce a higher level of NF-κB compared with that of the classical PRRSV strain CH-1a (*P* < 0.01).

Amino acid sequence alignment revealed that the Nsp2 of WUH3 and 07HBEZ possessed the same 30 amino acid discontinuous deletion [37]. Thus, we investigated whether these deletions have any effect on NF-κB activation. To test this hypothesis, we constructed several insertion mutants in Nsp2 expression plasmids based on the Nsp2 of PRRSV strain WUH3, which were denoted as Nsp2(+1), Nsp2(+29), and Nsp2(+1/+29). These mutants were then analysed for their capacities of stimulating NF-κB in HeLa cells. These assays showed that NF-κB activation by these Nsp2 insertion mutants was equivalent to that of the wild-type Nsp2 (Figure 4B) (*P* > 0.05). Additionally, insertions within this region did not result in the loss of the ability to activate NF-κB, suggesting that the 30 amino acids discontinuous deletion in the Nsp2 coding region is not related to NF-κB activation.

Taken together, the variable effect on Nsp2-induced NF-κB activation might be a factor contributing to the different levels of viral pathogenesis between different PRRSV strains. However, the underlying mechanism may be due to the different capacities of replication or the alternative strategies in NF-κB activation by different PRRSV strains. Using a series of chimeric viruses where the deletion region was substituted with corresponding regions of low virulence strains, Zhou *et al*. elucidated that the 30-aa deletion was not the primary determinant of virulence of HP-PRRSV. These findings are consistent with those in the current study that found there was no direct association between NF-κB activation and the 30-aa deletion in Nsp2. In addition, our investigation showed that Nsp2 specifically localized to the mitochondria (data not shown). Previous studies have demonstrated that the mitochondria played an important role in the activation of NF-κB and interferon regulatory factor (IRF)3 mediated by mitochondrial antiviral signalling (MAVS) in response to viral infection [38]. Whether the localization of Nsp2 in the mitochondria is essential for its capacity for NF-κB activation requires further investigation.

### Functional domain of Nsp2 for NF-κB activation

**Figure 5:**
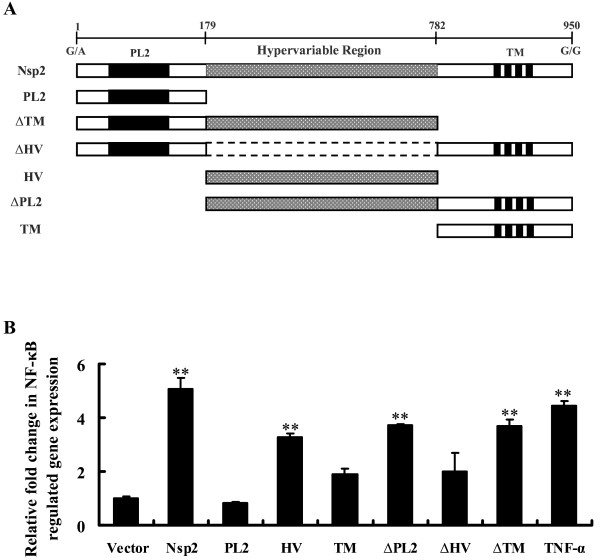
Characterization of the Nsp2 domains responsible for inducing NF-κB activation. (A) Schematic representation of various truncated mutants of PRRSV Nsp2 used in this study. PL2, the putative enzyme domain; HV, the hypervariable region; TM, the transmembrane region. (B) HeLa cells were co-transfected with the 0.2 μg of pNF-κB-Luc, 0.05 μg of pRL-TK, and the distinct truncations of Nsp2 expression plasmids (0.6 μg). An empty vector was used as a control. At 30 h after transfection, one group of cells with empty vector transfected was stimulated with 20 ng/mL of TNF-α. Cell extracts were collected at 36 h after the initial transfection and analysed for luciferase expression. **P < 0.01 as compared with vector control.

Since the 30-aa deletion in Nsp2 is not associated with its ability to activate NF-κB, we attempted to further map the domain responsible for in NF-κB activation. To this end, six truncated mutants of Nsp2 were constructed (Figure 5A) and a NF-κB reporter assay was performed to determine the functional region responsible for NF-κB activation. As shown in Figure 5B, several mutants, including the HV (hypervariable region), ∆PL2 (the putative enzyme domain truncated mutant), ∆TM (the transmembrane region truncated mutant), which all contain the HV domain, showed a significant increase in NF-κB luciferase activity. However, luciferase activity was significantly reduced in other mutants that did not contain these regions compared with wild-type Nsp2 (*P* < 0.01). This suggested that the functional domain responsible for NF-κB activation might be located in the middle HV of Nsp2.

Previous studies performed by Sun and co-workers [39] showed that the PRRSV Nsp2 OTU domain inhibited NF-κB activation by interfering with the polyubiquitination process of IκBα. These findings are consistent with our result that show the PL2 domain of PRRSV Nsp2 failed to activate NF-κB when expressed independently; however, the HV region of Nsp2 could activate NF-κB to similar levels as the full-length of Nsp2. This suggested that PRRSV Nsp2 is a multi-functional replicase subunit with different domains involved in different functions in viral replication and pathogenesis. In addition, the activation of NF-κB has been previously described in PRRSV-infected MARC-145 cells and alveolar macrophages [31,32]. However, recent studies have reported that several PRRSV Nsps, including Nsp1α, Nsp1β, Nsp11, could function as negative regulators of NF-κB to escape the host innate immune response [14,40]. Furthermore, studies from our laboratory showed that the N protein of PRRSV could induce NF-κB activation [41]. Taken together, these studies suggest that PRRSV might have developed sophisticated strategies for either activation or inhibition of NF-κB pathway to survive in host cells. Further investigation is required to evaluate the exact mechanisms involved in PRRSV-induced modulation of the NF-κB pathway.

## Conclusions

In summary, our data clearly demonstrated that the PRRSV Nsp2 alone, in the absence of other PRRSV proteins, could significantly activate the NF-κB pathway. Overexpression of PRRSV Nsp2 induced the NF-κB-dependent pro-inflammatory molecules, IL-6, IL-8, COX-2, and RANTES. The 30-aa deletion in the Nsp2 of HP-PPRSV was not associated with NF-κB activation; however, the hypervariable region of Nsp2 was essential for NF-κB activation. This study not only helps clarify the molecular mechanisms of NF-κB activation during PRRSV infection but also helps explain the ability of PRRSV Nsp2 to modulate the inflammatory response by regulating the NF-κB pathway. Understanding the relationships between PRRSV Nsp2 and the host inflammatory responses should help develop more effective strategies for the control of PRRS.

## Materials and methods

### Cells, viruses, and antibodies

HeLa and MARC-145 cells were cultured and maintained in Dulbecco’s modified Eagle medium (DMEM) supplemented with 10% heat-inactivated fetal bovine serum (FBS), 100 U/mL penicillin, 10 μg/mL streptomycin sulfate and then incubated at 37°C in a humidified 5% CO_2_ incubator. HP-PRRSV strain WUH3 (GenBank Accession No.: HM853673.2), a virulent strain, was isolated from the brain of a pig with the “high fever” syndrome in China at the end of 2006 and identified as a highly pathogenic North American type PRRSV [33]. PRRSV strain CH-1a (GenBank Accession No.: AY032626), the first field isolate in China, was kindly provided by Dr. Guangzhi Tong (Shanghai Veterinary Research Institute, Shanghai, China) [42]. Monoclonal antibody (mAb) anti-HA was purchased from Sigma (St. Louis, MO). Polyclonal antibodies (pAb), anti-β-actin, p65, p-p65, IκBα, were purchased from Santa Cruz (Santa Cruz, CA). Horseradish peroxidase-conjugated anti-mouse or anti-rabbit IgG antibodies were purchased from Beyotime Institute of Biotechnology (Jiangsu, China).

### Plasmids

Plasmids were constructed by standard methods. Expression plasmids of PRRSV nonstructural proteins used in this report were constructed by RT-PCR amplification from the genomic RNA of PRRSV strain WUH3. Mutagenesis of 1 or/and 29 amino acids in Nsp2 was performed using the Overlap Extension Polymerase Chain Reaction. Six truncated mutants of Nsp2 were generated by PCR from WUH3 cDNA. All PCR amplification reactions were performed by PrimerSTAR™ HS DNA Polymerase system (TaKaRa Biotechnology, Dalian, China). The details of the specific primers used in PCR steps are available on request.

Gene fragments were cloned into the pCAGGS-HA vector in-frame that was constructed using the pCAGGS vector (Stratagene, Santa Clara, CA) to generate a series of PRRSV nonstructural protein constructs and Nsp2 mutants for expression in mammalian cells. All constructs were confirmed by sequencing and the expressions of the interested protein were verified by western blot analysis using anti-HA antibody (data not shown). The expression plasmid of Nsp2 of PRRSV 07HBEZ strain (GenBank Accession No.: FJ495082.2 and HM595639) was kindly provided by Dr H. X. Li [43]. Full-length Nsp2 of CH-1a strain was amplified from the cDNA of CH-1a. The luciferase reporter plasmid pNF-κB-Luc contains four repeats of κB binding motifs followed by the luciferase reporter gene (Luc) and the internal control plasmid pRL-TK were purchased from Stratagene. Other reporter plasmids, such as IL-6-Luc, IL-8-Luc, RANTES-Luc, and COX-2-Luc, were constructed as previously described [34,44]. The p65 gene was derived from human RelA cDNA and cloned into the pEGFP-C1 vector. The DNA fragment containing the entire open reading frame (ORF) of Nsp2 was excised from pCAGGS-HA-Nsp2 and inserted into the pDsRed-C1 (Clontech, Mountain View, CA), resulting in the expression construct pDsRed-Nsp2.

### Transfection and luciferase reporter assay

Transient transfection was performed using Lipofectamine 2000 (Invitrogen). HeLa or MARC-145 cells were seeded on 24-well plates (Nunc, Roskilde, Denmark) at a density of 2–4 × 10^5^ cells/well and cultured until the cells reached approximately 70–80% confluence, and were then transfected with the indicated plasmids. For each transfection, 0.2 μg of the reporter plasmid (NF-κB, IL-6, IL-8, COX-2, RANTES-Luc) along with 0.05 μg of pRL-TK for normalization and 0.6 μg of various expression plasmids or empty control plasmid (pCAGGS-HA) were used. In addition to serving as a negative control, the empty vector was also used to adjust the total amount of transfected DNA to 1.05 μg in dose-dependent experiments. Cells treated with 20 ng/mL of TNF-α (Sigma) for 6 h prior to harvest were used as a positive control. Cell extracts were collected at the indicated time points. Firefly and Renilla luciferase activities were measured using the Dual-luciferase reporter assay system (Promega, Madison, WI) in a MicroBeta TriLux liquid scintillation and a luminometer (Turner BioSystems, Sunnyvale, CA), according to the manufacturer’s instructions.

### Cellular extracts and western blot assay

Cytoplasmic and nuclear protein extracts from HeLa cells after transfection with Nsp2-expression plasmid were prepared with the Cytoplasmic Extraction Reagent (100 mM HEPES [pH 8.0], 1% NP40, 15 mM MgCl_2_, 100 mM KCl, 5 mM DTT, 2 mM Sucrose, 1% Cocktail) and Nuclear Extraction Reagent (200 mM HEPES [pH 7.9], 15 mM MgCl_2_, 4.2 mM NaCl, 5 mM DTT, 5 mM EDTA, 1% Cocktail), respectively. Protein concentration was determined by Micro BCA Protein Assay (Thermo, Rockford, IL) with bovine serum albumin as a standard following the manufacturer’s protocols. The extracts were boiled in sodium dodecyl sulphate (SDS) protein sample buffer (2% SDS, 60 mM Tris–HCl [pH 6.8], 10% glycerol, 0.001% bromophenol blue, and 0.33% β-mercaptoethanol), then resolved by 10% acrylamide sodium dodecyl sulphate polyacrylamide gel electrophoresis (SDS-PAGE). The separated proteins were electroblotted onto a PVDF membrane (Millipore, Billerica, MA). Membranes were blocked with 5% (w/v) dried skim milk in tris-buffered saline containing Tween 20 (TBST). Western blotting was performed using a standard techniques, using anti-HA monoclonal antibody, anti-p65, p-p65, IκBα and β-actin polyclonal antibodies at a dilution of 1:1,000. Secondary antibodies, horseradish peroxidase-conjugated anti-mouse and anti-rabbit IgG antibody, were used at a dilution of 1:2,500, and protein bands were visualized using SuperSignal West Pio Luminol kit (Pierce).

### Confocal fluorescence microscopy

HeLa cells were seeded on microscope coverslips placed in 24-well plates at a concentration of 2 × 10^5^ cells/well until the cells reached approximately 70–80% confluence. The pEGFP-p65 was co-transfected with pDsRed1-Nsp2, as well as the empty vector pDsRed-C1. At 36 h post-transfection, cells were fixed with 4% paraformaldehyde for 10 min followed by permeabilization with 0.1% Triton X-100 for 10 min at room temperature (RT). After three washes with PBS, cells were incubated with DAPI for 5 min at room temperature. After washing with PBS, fluorescent images were performed using confocal laser scanning microscope (LSM 510 Meta; Carl Zeiss MicroImaging, Göttingen, Germany).

### RNA exaction and quantitative real-time RT-PCR

To determine the effect of Nsp2 on IL-6, IL-8, COX-2 and RANTES, HeLa cells grown in 24-well plates were transfected with 1 μg of plasmid encoding Nsp2. The empty vector served as a negative control. Total RNA were extracted from the transfected cells 36 h post-transfection using TRIzol reagent (Invitrogen). Real-time RT-PCR was performed using SYBR Green Real Time PCR Master Mix (Toyobo Biologics, Osaka, Japan) with primer pairs described in Table 1 in the LightCycler 480 (Roche Molecular Biochemicals). Individual transcripts in each sample were assayed three times and normalized with human glyceraldehyde-3-phosphate dehydrogenase (GAPDH) mRNA level as an endogenous control. Primers were designed using the Primer Express software (version 3.0; Applied Biosystems, Carlsbad, CA).

**Table 1:** Primers used in the quantitative real-time PCR

**Primer names**	**Sequences(5′-3′)**
RANTES	F: AGCCCTCGCTGTCATCCTG
	R: GGGCAATGTAGGCAAAGCAG
IL-6	F: AGAGGCACTGGCAGAAAAC
	R: TGCAGGAACTGGATCAGGAC
IL-8	F: ACTCCAAACCTTTCCACCCC
	R: TTCTCCACAACCCTCTGCAC
COX-2	F: CCCTCAGACAGCAAAGCCTA
	R: GGTGGGAACAGCAAGGATT
GAPDH	F: CGGGGCTCTCCAGAACATC
	R: CTTCGACGCCTGCTTCAC

### Statistical analysis

All experiments were performed at least three times with reproducible results. Data were presented as means ± standard deviations (SD). The Student’s *t*-test was used to determine statistical significance. *P*-values less than 0.05 were considered statistically significant, and *P*-values less than 0.01 were considered highly significant.

## Competing interests

The authors declare that they have no competing interests.

## Authors’ contributions

YF was responsible for carrying out the experiments, interpretation of data, and drafting the manuscript. SBX, LRF and HCC participated in the design of the study and contributed to write the manuscript. YW, DW performed the statistical analysis. RL, YYL participated in the plasmid construction. All authors read and approved the final manuscript.
